# Supplementary material for: Symmetry-broken Josephson junctions and superconducting diodes in magic-angle twisted bilayer graphene
Source: Nat Commun. 2023 Apr 26;14:2396. doi: 10.1038/s41467-023-38005-7 (PMC10133447; doi:10.1038/s41467-023-38005-7)
Supplement: Supplementary file 1 — Supplementary information [file 41467_2023_38005_MOESM1_ESM.pdf]

Supplementary Information for ‘Symmetry-broken Josephson junctions and superconducting diodes in magic-angle twisted bilayer graphene’

J. Díez-Mérida[1], A. Díez-Carlón[1], S. Y. Yang[1], Y.-M. Xie[2], X.-J. Gao[2], J. Senior[4], K. Watanabe[3], T. Taniguchi[3], X. Lu[1], A. P. Higginbotham[4], K. T. Law[2] and Dmitri K. Efetov[1]*

1. ICFO - Institut de Ciencies Fotoniques, The Barcelona Institute of Science and Technology, Castelldefels, Barcelona, 08860, Spain
2. Department of Physics, Hong Kong University of Science and Technology, Clear Water Bay, Hong Kong, China
3. National Institute of Material Sciences, 1-1 Namiki, Tsukuba, 305-0044, Japan
4. IST Austria, Am Campus 1, 3400 Klosterneuburg, Austria

*E-mail: dmitri.efetov@physik.lmu.de

Supplementary Discussion

A. Fabrication and transport characterization of device A.

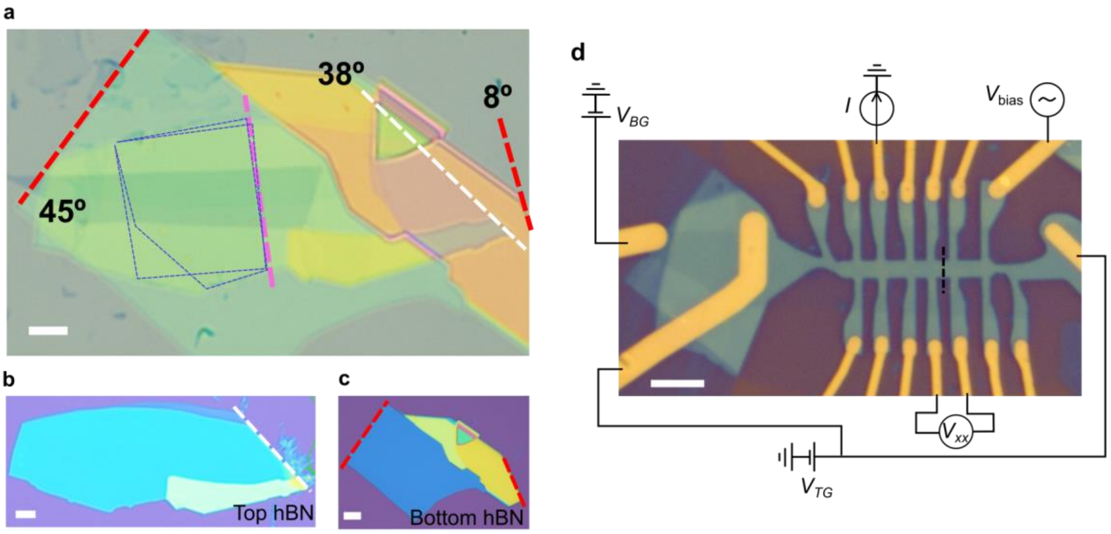

Supplementary Fig. 1. Optical image of the device and alignment to hBN. a, Resulting final stack, where the red and white dotted lines show misalignment between the edges of top and bottom hBN and those of the two graphene flakes composing the MATBG in blue. **b – c,** Optical micrographs of top and bottom hBN used for stacking the van-der-Waals heterostructure shown in **a**. The white and red dotted lines mark the crystallographic edges of the two hBN flakes. **d,** Optical picture of the final device, with a schematic of our 4-probe measurement setup, where V_{bias} is the source voltage, I is the current through the device, V_{xx} the voltage drop between the measurement probes and V_{BG} (V_{TG}) correspond to the back (top) gate voltage. The black dotted line marks the position of the narrow-etched region of the top-graphite gate, corresponding to the location of the weak link in the junction. Scale bars equal 5 µm in all figures.

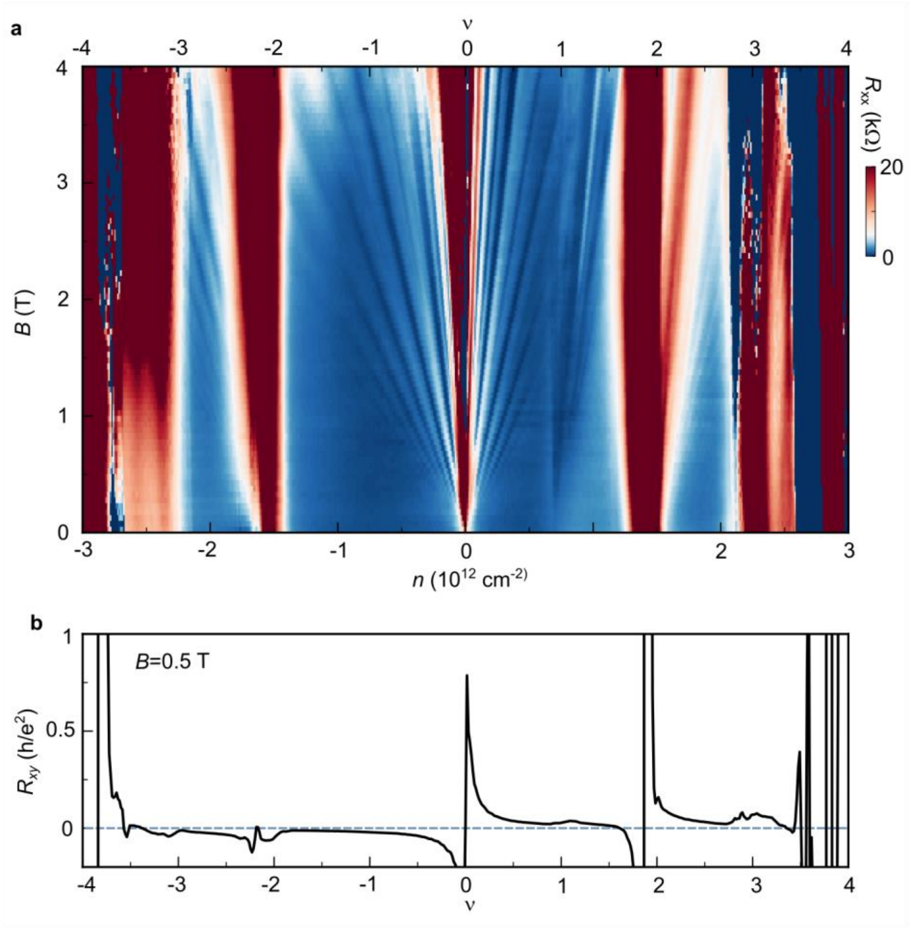

Supplementary Fig. 2. Landau fan diagram and absence of quantized plateau Hall resistance at low field. a, Longitudinal resistance R_{xx} vs. carrier density *n* and perpendicular magnetic field *B*, where Landau levels were used to calculate the twist angle of the device. **b,** Anti-symmetrized transverse resistance R_{xy} vs. *n* at *B* = 0.5 T, plotted in units of h/e^2, where *h* is Planck's constant and *e* is the electron charge. No quantized plateau Hall resistance close to υ = -2 is observed. All data in the figure is taken setting V_{TG} = 0 V.

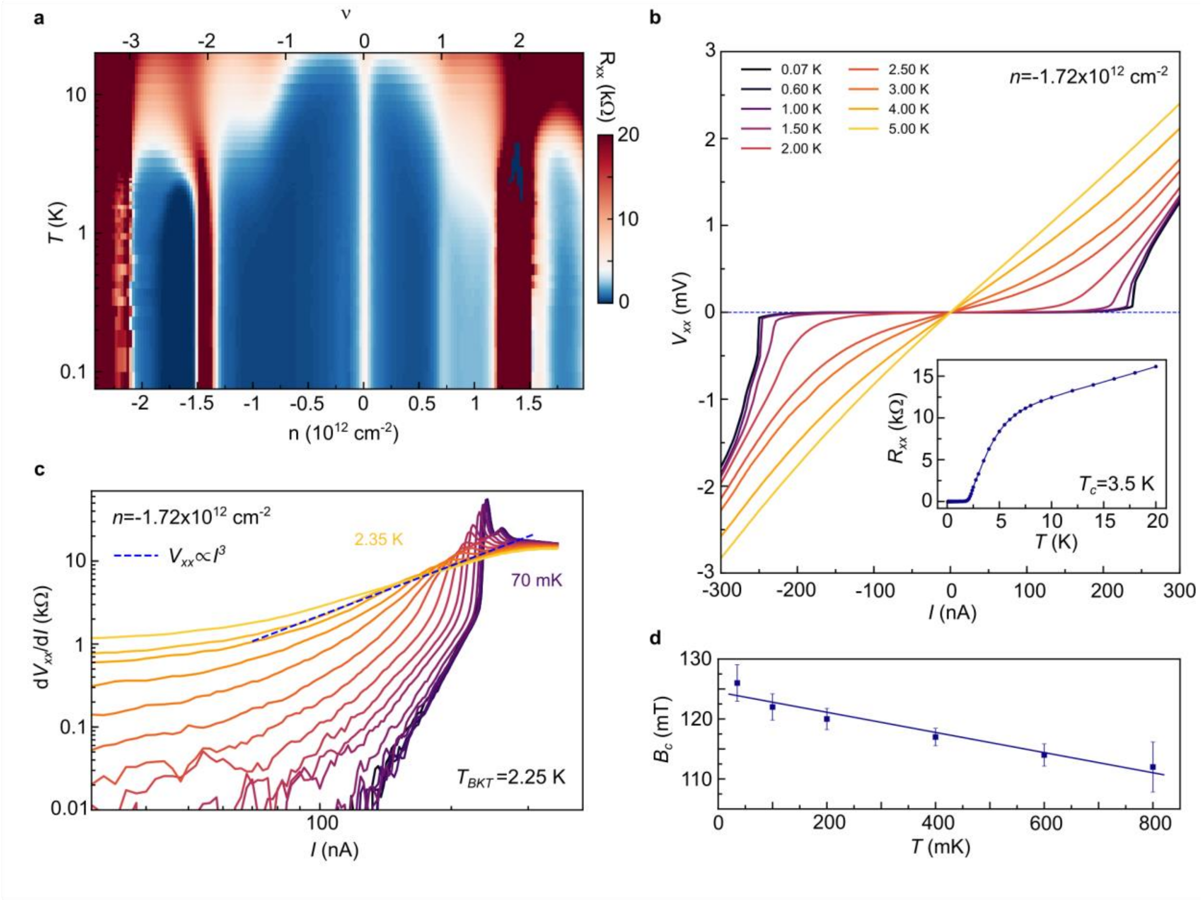

Supplementary Fig. 3. Temperature dependence and characterization of the superconducting state. a, Longitudinal resistance R_{xx} as a function of carrier density n and temperature T. **b,** Current-voltage (I-V) characteristic of the superconducting state at $n = -1.72 \times 10^{12}$ cm^{-2} for a wide range of temperatures. From the lowest temperature data, we extract a critical current of I_c = 240 nA. The inset shows R_{xx} as a function of T, where a transition to the superconducting state is observed with a critical temperature T_c = 3.5 K, taken as the temperature at which R_{xx} equals to 50% of the normal state resistance. **c,** Differential resistance dV_{xx}/dI vs. d.c. current I at various temperatures. Fitting $V_{xx} \propto I^3$ yields a BKT transition temperature T_{BKT} = 2.25 K. **d,** Perpendicular critical field B_c vs T taken as half of the normal state resistance values. From linearly fitting $B_c = (\Phi_0/2\pi\xi_{GL}^2)(1 - T/T_c)$, we extract a Ginzburg-Landau superconducting coherence length $\xi_{GL} = 106 \pm 8$ nm at T = 0, where $\Phi_0 = h/2e$ is the superconducting flux quantum. All data in the figure is taken setting V_{TG} = 0 V.

B. Dual gate characterization of device A and electrostatic model.

Supplementary Fig. 4a shows R_{xx} as a function of back gate voltage V_{BG} and top gates voltage V_{TG}, measured at 1.8 K. This dual-gate map contains two distinct features, corresponding to the global carrier density n and junction carrier density n_J regions sketched in the inset of Fig. 1b of the main text. The diagonal features, marked as gray dashed lines in the figure, are those gated both by the back gate and the top gates. Its carrier density is given by $n = C_{BG}V_{BG} + C_{TG}V_{TG}$, where C_{BG} and C_{TG} are the capacitances of the back gate and top gates, respectively. The features corresponding to the junction are displayed as green dashed lines. This region is mainly gated by the back gate, but having a small contribution from the top gates due to the effect of stray fields[1]. This explains why these features are not perfectly vertical but rather have a finite slope. The carrier density of this region follows $n_J = C_{BG}V_{BG} + \alpha C_{TG}V_{TG}$, where $\alpha \ll 1$. As such, we can independently gate the two regions.

Supplementary Fig. 4b is a zoom-in of the dual-gate map measured at 35 mK, where the dark blue region corresponds to the superconducting state. By following this dark blue region parallel to the grey lines, the dual-gated main regions remain in the superconducting phase, whereas the single-gated junction region can be continuously changed in doping, creating different configurations of a JJ, as indicated by the white squares. This diagonal line is the one followed to take the dV_{xx}/dI colormap of Fig. 1d in the main text.

In order to gain insight on how the electrostatics of the dual gated architecture of our device works, we have performed electrostatic simulations by solving the Poisson equation with a finite difference method[2]. The generalized Poisson equation is given by $\nabla \cdot [\epsilon(\boldsymbol{r})\, \nabla V(\boldsymbol{r})] = \rho(\boldsymbol{r})/\epsilon_0$, where ϵ is the dielectric constant (ϵ = 4 for hBN), V is the electrostatic potential, ρ is the density of electric charges and ϵ_0 is the vacuum permittivity. The problem is solved self-consistently with an iterative approach following a successive over-relaxation method[3]. Dirichlet boundary conditions (BCs) are used for the electrodes: $V(z = 20\text{ nm}) = V_{TG}$ and $V(z = -20\text{ nm}) = V_{BG}$. The sides and top regions with no gate are set with Neumann BCs, satisfying $\partial V_x = 0$ and $\partial V_y = 0$, respectively. The model is strictly electrostatic, not including any band structure properties of the MATBG. Supplementary Fig. 5a shows the response of the case where the weak link is set very close to the $\nu = -2$ CI (SC/CI'/SC configuration). The linecut shown in Supplementary Fig. 5b is extracted from the dashed gray line of the figure, where the MATBG would be located. Here the electrostatic potential is converted to n by a factor related to the capacitances of the top and back gates. From the simulations it is possible to estimate the effective size of the JJ (d_J), which does not match the length of the etched region, $d \approx 150$ nm. Since n has a slow transition from the SC to the CI state, the effective length of the JJ is $d_J \approx 100$ nm.

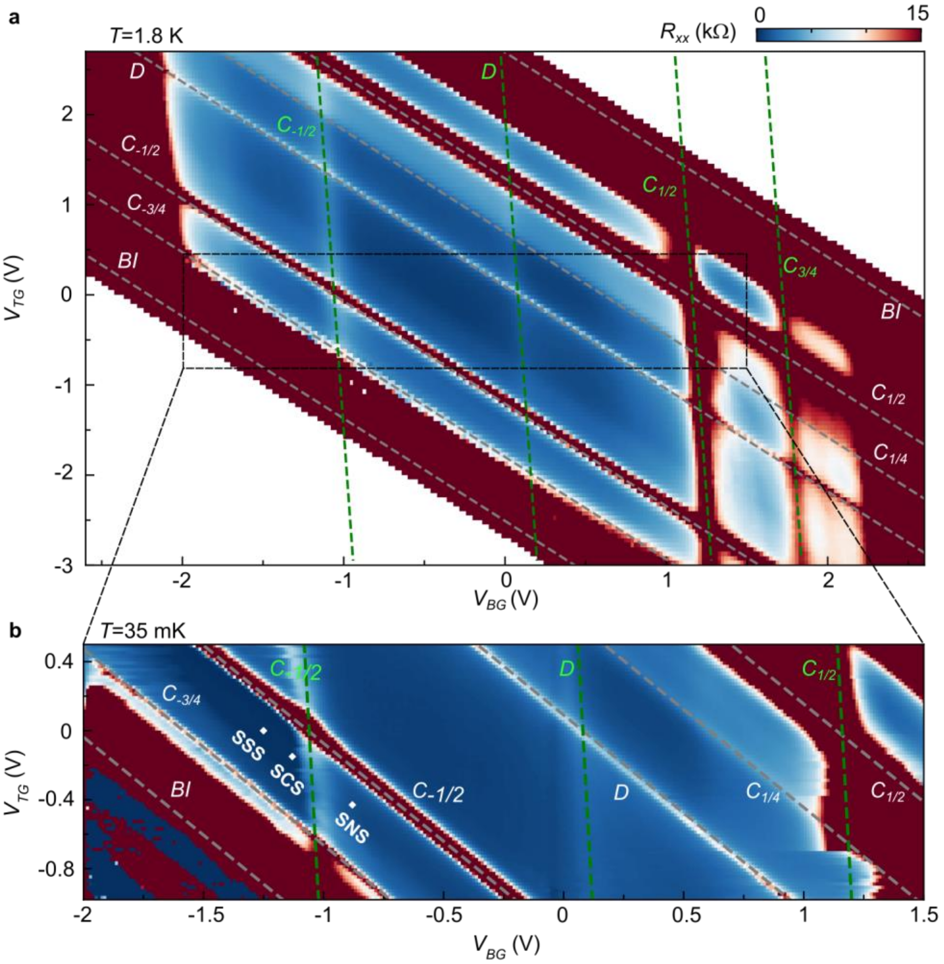

Supplementary Fig. 4. Dual-gated maps. a, Longitudinal resistance R_{xx} vs. back gate voltage V_{BG} and top gate voltage V_{TG} at T = 1.8 K. The diagonal features marked with grey dashed lines are the integer fillings of the main regions (n in Fig. 1b of main text), gated by both the back gate and top gates. Slightly tilted vertical features, fitted with green dotted lines, are the integer fillings of the junction region (n_J in Fig. 1b of main text), mainly gated by the back gate. Labels of the integer filling factors marked by dashed lines correspond to: band insulator (*BI*) at full filling, correlated insulator at plus/minus three-quarter filling ($C_{\pm 3/4}$), plus/minus half filling ($C_{\pm 1/2}$) and one quarter filling ($C_{1/4}$) and Dirac point (D). **b,** Zoom-in of black-delimited area in **a** taken at base temperature T = 35 mK, where the superconducting state is fully developed. White squares mark the diagonal line in the map at which both main regions are kept in the superconducting state, and the junction region is set at different doping.

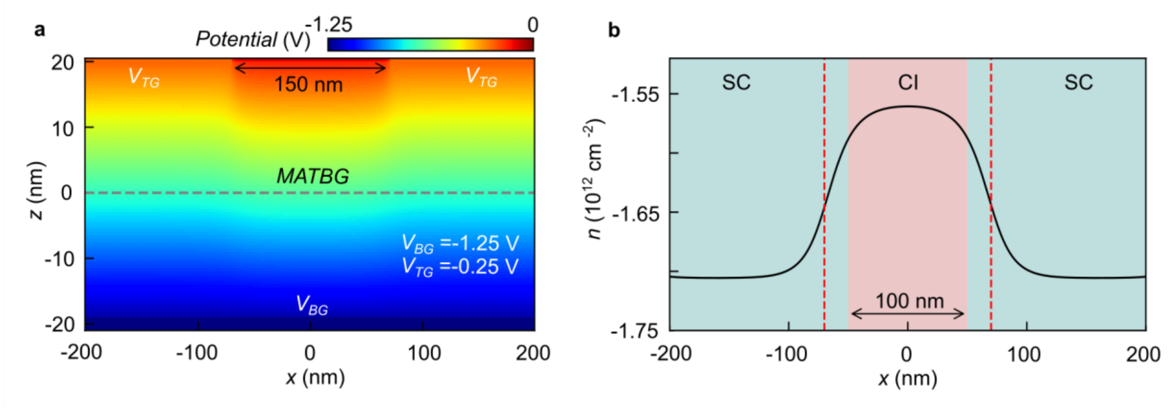

Supplementary Fig. 5. Simulations of the electrostatic potentials governing our devices. a, Calculated potentials as a function of position *x* and height *z*, for a junction configuration with back gate voltage V_{BG} = –1.25 V and top gate voltage V_{TG} = –0.25 V. The back gate and top gates are placed at *z* = –20 nm and *z* = 20 nm, respectively. The etched region is marked by the double arrow at the top of the figure. The dashed gray line shows the position at which the MATBG would be located. **b,** Extracted linecut of the electrostatic potential from **a** at *z* = 0 nm, the MATBG position. Vertical red dashed lines show the position where the top graphite was etched. The light green and red regions show the extension of the superconducting (SC) and correlated insulating (CI) states. The effective length of the JJ in this case is 100 nm, as marked in the figure.

C. Device B characterization

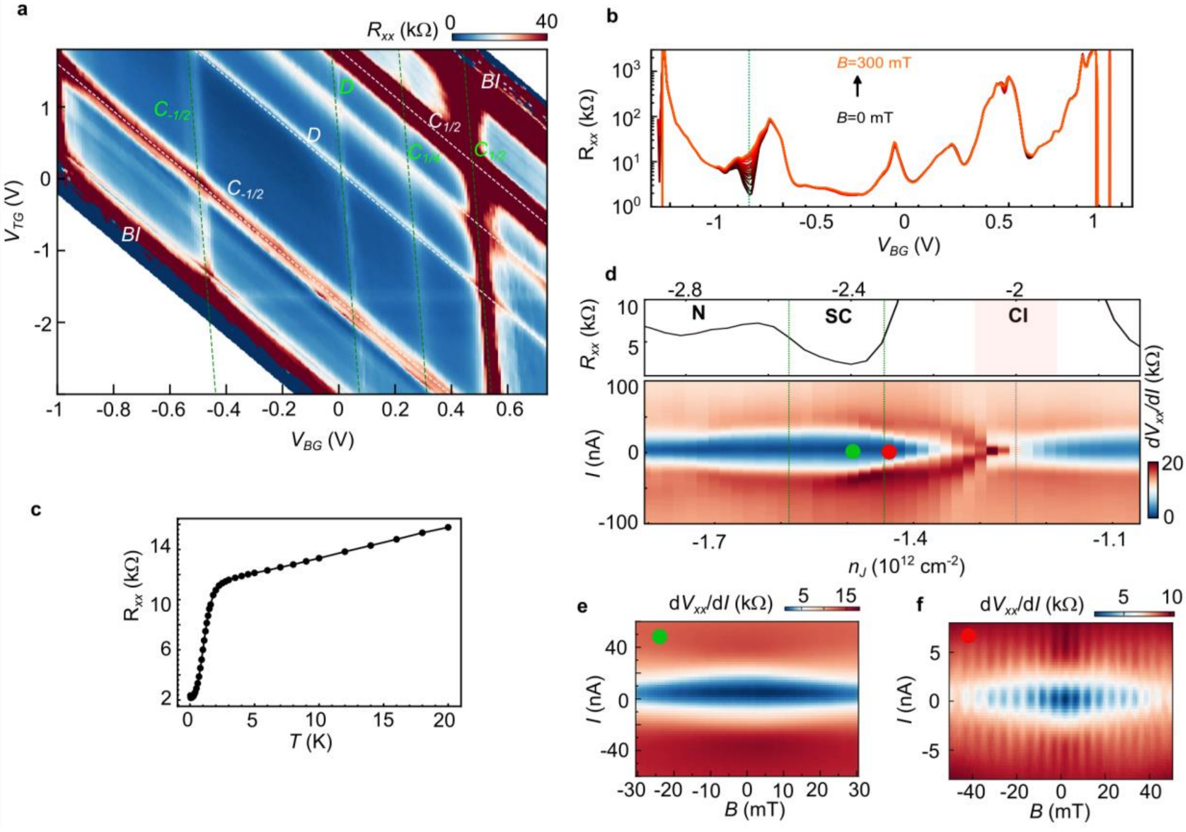

Supplementary Fig. 6. Device B transport characterization. **a,** Longitudinal resistance R_{xx} vs. back gate voltage V_{BG} vs. top gate voltage V_{TG} at temperature T = 35 mK. Very similar features to device A are visible and highlighted with the same nomenclature as in Supplementary Fig. 4: band insulator (*BI*) at full filling, correlated insulator at plus/minus half filling ($C_{\pm 1/2}$) and one quarter filling ($C_{1/4}$) and Dirac point (*D*). **b,** R_{xx} vs. filling factor ν vs. perpendicular magnetic field B at low fields. **c,** R_{xx} vs. T characteristics of the SC at the center of the dome as marked by the green dashed line of **b**. **d,** (Top) Magnification of **b** around the superconducting (SC) state $-3 < \upsilon < -1.8$. (Bottom) Differential resistance dV_{xx}/dI vs. d. c. current I at different junction carrier densities n_J, keeping the global carrier density $n = -1.44\times10^{12}$ cm^{-2} in the SC state. Dashed green vertical lines mark the position where n_J is no longer in the SC state. N and CI correspond to the normal metal and correlated insulator state, respectively. The colored dots mark the position where the Fraunhofer patterns of **e** and **f** are measured. **e,** Fraunhofer pattern of the SC/SC/SC position in this device. **f,** Fraunhofer pattern of the SC/CI'/SC position in this device.

D. Evolution of the asymmetric Fraunhofer patterns with n and T, and I_c extraction

Supplementary Fig. 7a displays an extension of the dV_{xx}/dI vs I map shown in Fig. 1d of the main text, to better identify the n_J positions of the Fraunhofer patterns, which are labelled with color dots. Supplementary Fig. 7b-e show the evolution of the Fraunhofer patterns when the weak link is being doped at different carrier densities n_J. Upon tuning n_J from -1.72×10^{12} cm^{-2} (SC/SC/SC configuration) to -1.56×10^{12} cm^{-2} (SC/CI'/SC configuration), the Fraunhofer evolves from being symmetric in both current and field towards being asymmetric. When $n_J \gtrsim -1.35\times10^{12}$ cm^{-2} or $n_J \lesssim -2.1\times10^{12}$ cm^{-2}, the coherence is lost in our JJ and so we do not observe a supercurrent anymore. However, distinct superconducting non-linearities remain, which are in line with Andreev reflections at the SC interfaces[4].

To study the behavior of the JJ with magnetic field, a detailed analysis of I_c at different currents and fields is performed in the main text (Fig. 3 and 4). The exact procedure by which we define and extract I_c is shown in Supplementary Fig. 8a. We define I_c as the I value at which the resistance is non-zero anymore. We define a R threshold value of $\approx$100 Ω, displayed as the dashed red line and I_c is taken as the I value at which the dV_{xx}/dI exceeds such a threshold, marked by the red cross in the figure. Since this procedure is highly dependent on the value of the defined threshold, several values are considered, and an error is taken by calculating the standard deviation of all the extracted I_c values. The error obtained from this analysis is the error which is plotted in the temperature dependence of ΔB in Fig. 4. The main value of I_c is chosen as the best fit to the contour in the Fraunhofer pattern, as shown in Supplementary Fig. 8b. In this example we plot the 35 mK data shown in Fig. 1e (right) of the main text. The extracted I_c^+ and I_c^- are plotted as a red and green dashed line over the map. The same procedure is followed for all the Fraunhofer patterns mentioned in the main text.

Finally, in this section, we discuss the temperature dependence of the Fraunhofer pattern of the SC/CI'/SC configuration and its magnetic hysteresis. Supplementary Fig. 9 shows the SC/CI/'SC Fraunhofer patterns measured at different temperatures that are not shown in the main text. For every temperature, the Fraunhofer is measured with field sweeping in both directions. The insets of each of these plots demonstrate the fact that the position and magnitude of coherence peaks is reversed at zero field for opposite field sweeping directions, due to the hysteretic behavior inside the JJ. The hysteresis is further evidenced in the I_c vs. B plots (Supplementary Fig. 9 l-p) where $|I_c^+(B_{\mathrm{up}})| \neq |I_c^+(B_{\mathrm{down}})|$, and is gradually lost until T = 800 mK, where $|I_c^+(B_{\mathrm{up}})| = |I_c^+(B_{\mathrm{down}})|$. The temperature dependence of ΔB shown in Fig. 4d of the main text is extracted from this data.

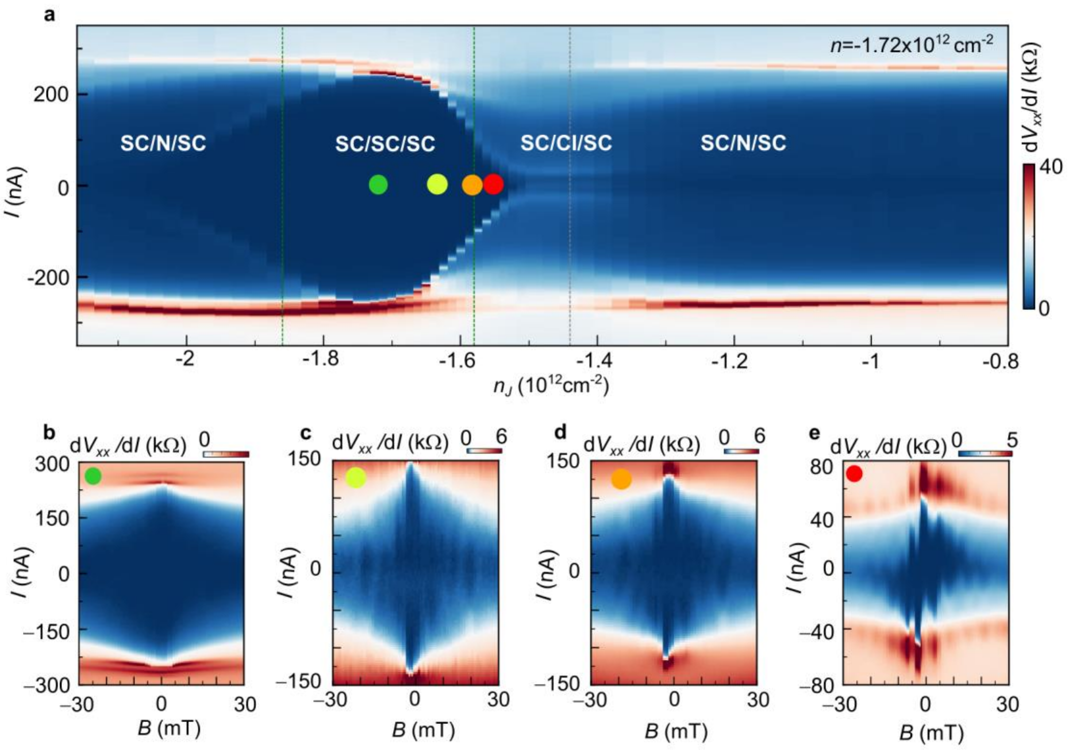

Supplementary Fig. 7. Carrier density dependence of the asymmetry in the Fraunhofer patterns. a, Differential resistance dV_{xx}/dI vs. d. c. current I and junction carrier density n_J at T = 35 mK, keeping the global carrier density n in the superconducting state. SC, N and CI label the superconducting, normal metal and correlated insulator state. **b-e,** Fraunhofer patterns with the weak link being at different doping n_J, corresponding to those labelled by the colored circles in **a**. The Fraunhofer patterns evolve from being symmetric in the SC/SC/SC to being asymmetric in the SC/CI'/SC configuration.

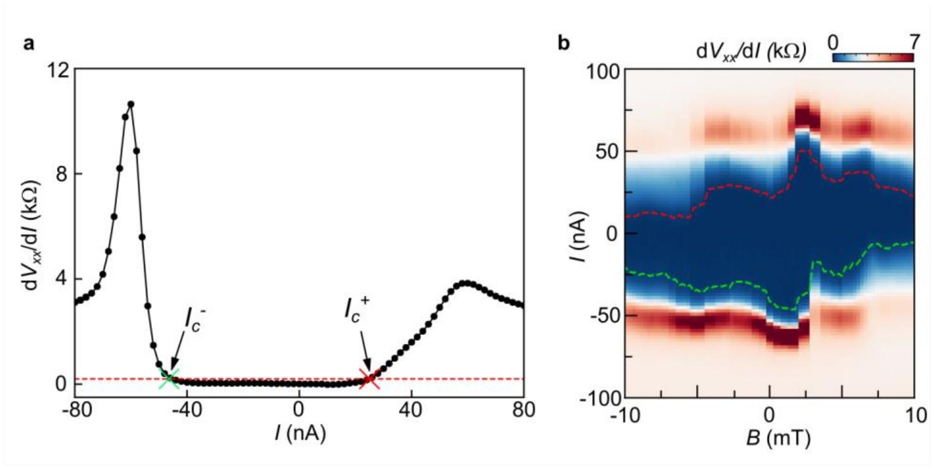

Supplementary Fig. 8. Critical current I_c extraction. The plotted data corresponds to the same Fraunhofer pattern as in Fig. 1e (right) of the main text. **a,** Differential resistance dV_{xx}/dI vs. d. c. current I linecut of **b** at zero field. The red dashed line marks a threshold value defining the end of the superconducting state, from which the positive and negative critical currents (I_c^+ and I_c^-) are extracted. **b,** dV_{xx}/dI vs. I and perpendicular magnetic field B. The extracted I_c^+ and I_c^- are sketched as dashed red and green lines.

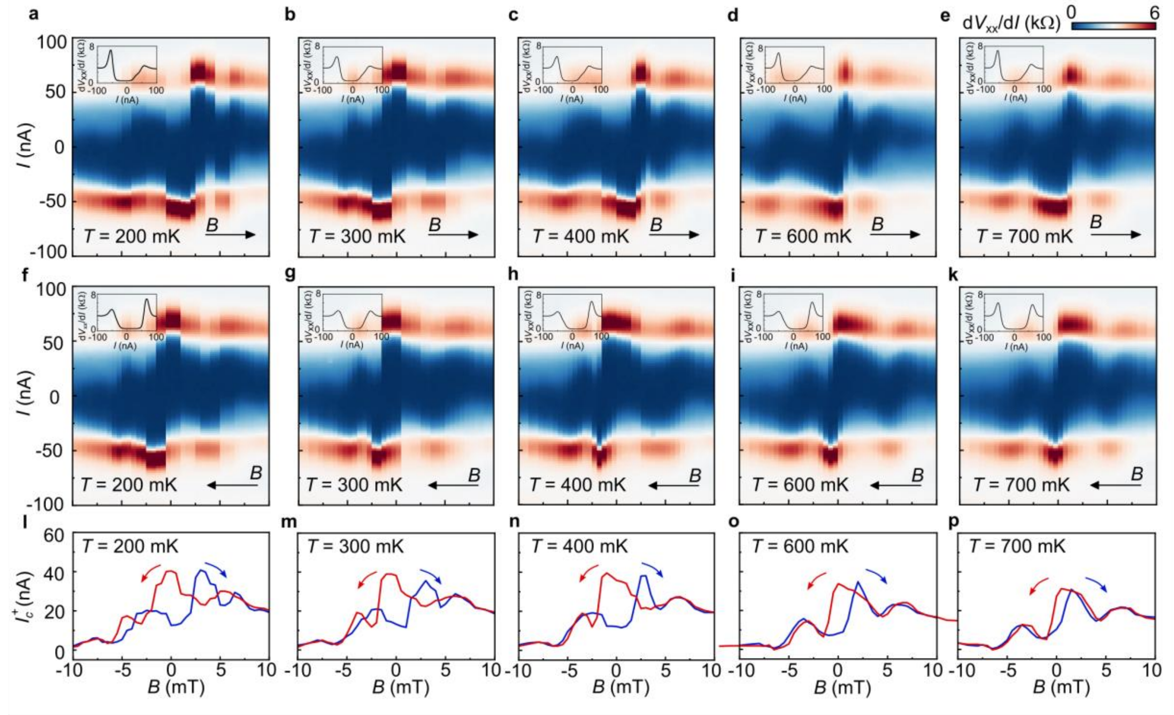

Supplementary Fig. 9. Temperature dependence of the SC/CI'/SC Fraunhofer pattern and its hysteresis. a-e, Fraunhofer patterns measured at junction carrier density n_J = -1.56×10^{12} cm^{-2}, at increasing temperatures T from 35 mK to 700 mK and by sweeping the magnetic field up, as marked with the black arrow. Insets of each plot show a differential resistance dV_{xx}/dI vs. d.c. current I linecut at perpendicular magnetic field B=0 T. **f-k,** Corresponding Fraunhofer patterns to **a-e** respectively, at the same temperature but opposite magnetic field sweep direction. **l-p,** Extracted positive critical current I_c^+ from the corresponding temperatures and with field sweeping up (blue) or down (red).

E. Disentangling different magnetic signals

A key finding of the results is that we observe two distinct magnetic states. The effect that survived to the T_c of the JJ is attributed to orbital magnetism, while the effect found only at the lowest T is discussed in different possible scenarios. Apart from the T, the main way to distinguish these effects is through their behavior with magnetic field. While the orbital magnetization requires fields higher than ~300 mT to be polarized, the other state can be polarized with fields of ~3 mT. This distinction is shown in Supplementary Fig. 10. Supplementary Fig. 10a shows the dV_{xx}/dI measured at B = 0 mT and T = 35 mK right after cool-down from 20 K to get rid of all the magnetization effects. When no pre-magnetizing field B_M has been applied (B_M = 0 mT), the dV_{xx}/dI is rather symmetric with respect to I (black curve). After applying a $B_M \leq$ 3 mT, the curves remain symmetric, while for $B_M \geq \pm$ 5 mT, the asymmetry appears. Supplementary Fig 10b shows the same measurement but now at 800 mK. First, the sample is warmed up to 20 K, cooled down and measured at 800 mK without applying any field (black curve). Then we see how for B_M = ± 50 mT the curves remain the same, while for $B_M \geq \pm$ 300 mT the asymmetric I_c appears. A further difference is the direction of the asymmetry. For the low T state, applying B_M > 0 makes $I_c^+ > |I_c^-|$, while the opposite is true for the orbital magnetic state.

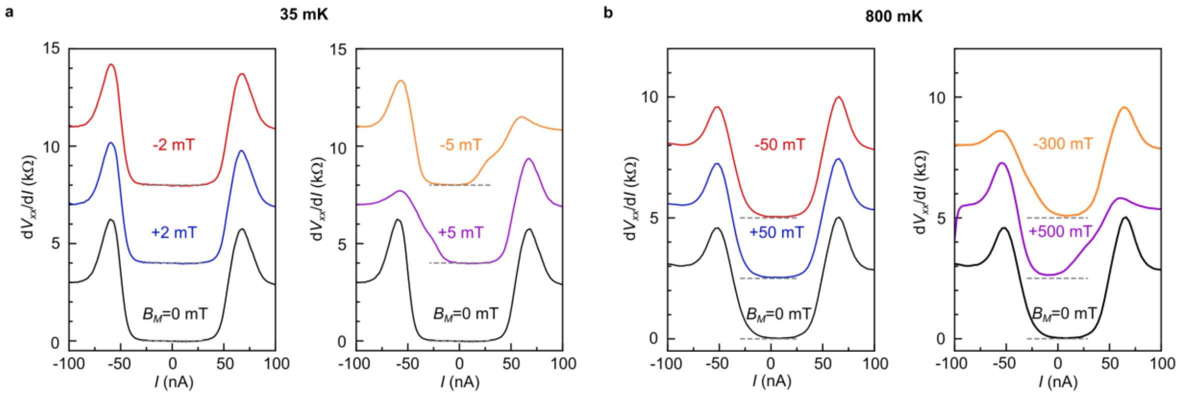

Supplementary Fig. 10. Distinguishing between high and low field hysteresis. Differential resistance dV_{xx}/dI measured right after cooling down (black curves) or after applying different pre-magnetizing fields B_M at temperature T = 35 mK **a,** and T = 800 mK **b**. While at 35 mK, fields above 3 mT are enough to switch the magnetic state, at 800 mK, fields above 300 mT are needed, proving we have two distinct effects. The behavior of the dV_{xx}/dI is also different for the two effects, i.e. for the low field effect $I_c^+ > |I_c^-|$, when $B_M > 0$, whereas the opposite is true for the high field effect. This is clear comparing the orange and purple curves at the two temperatures.

F. Demonstrating the superconducting diode effect.

We demonstrate the superconducting diode behavior in Supplementary Fig. 11. In Supplementary Fig. 11b we apply $|I| \sim 25$ nA and continuously switch the current direction. Simultaneous measurements of the device resistance show that the device is clearly resistive in one current direction and superconducting in the other, depending on the magnetization direction.

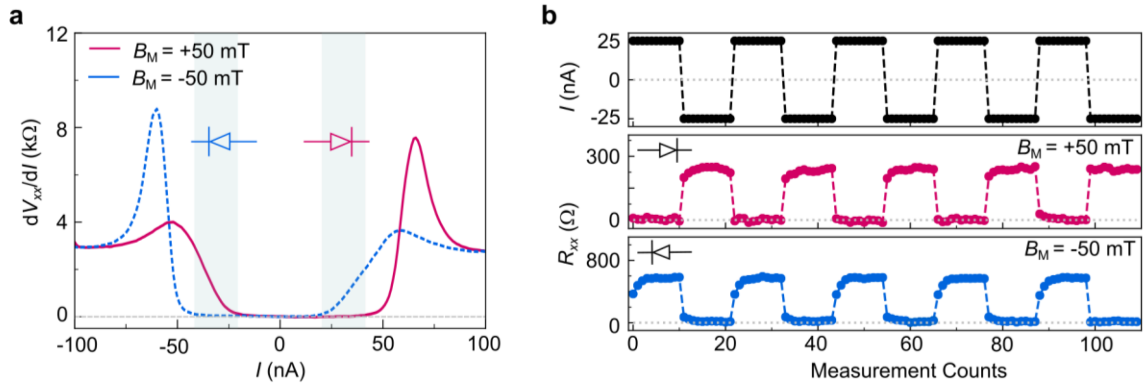

Supplementary Fig. 11. Zero-field switchable superconducting Josephson diode. a, dV_{xx}/dI vs. I measured setting n_J = -1.56×10^{12} cm^{-2} at 35 mK. All curves are taken at B=0 mT after pre-magnetizing the sample at B_M = +50 mT (red) or B_M = -50 mT (blue). The shaded gray regions mark the values of current at which the diode behavior is observed. **b,** Switching between resistive and superconducting state by changing the direction of I as shown in the top panel. By applying opposite B_M, the diode behavior is inverted (red and blue curves).

G. Two-dimensional planar and three-dimensional bulk behavior of the Fraunhofer patterns

In a two-dimensional superconductor, the film thickness is smaller than the London penetration depth λ. In this case, the magnetic flux can penetrate into the superconductor and the spatial distribution of magnetic field is governed by the Pearl length $\Lambda = 2\lambda^2/t$, where t is the film thickness [5]. It has been previously shown[6,7] that the critical current of a two-dimensional JJ under a perpendicular magnetic follows the relation $\Delta B_{2D} \approx 1.8\,\Phi_0/w^2$, where w is the lateral size of the junction. This is different from a three-dimensional bulk Josephson junction[8], where the period of the oscillations follows $\Delta B_{3D} \approx 1.8\,\Phi_0/wd$ where d is the length of the junction. According to our JJ geometry ($w \approx 1.2 \pm 0.1$ µm and $d_J \approx 100$ nm), ΔB_{3D} is estimated to be ~ 16 ± 1 mT, which is far larger than ΔB_{2D} ~ 2.5 ± 0.5 mT, as shown in the calculations in Supplementary Fig. 12. The modelled data of section H are calculated considering the 2D JJ formalism for the description of the flux.

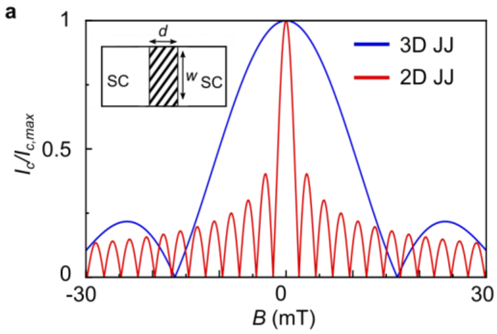

Supplementary Fig. 12. Planar and bulk Josephson Junctions Fraunhofer patterns. a, Calculations of the expected normalized critical current $I_c/I_{c,max}$ vs. perpendicular magnetic field B for a JJ of our sample dimensions ($w \approx 1.2$ µm and $d \approx 100$ nm, where w and d are the width and length of the junction) in the 2D planar regime (red) and the 3D bulk regime (blue).

H. Modeling Fraunhofer patterns from Real Space Current Density Distribution

The critical current of a Josephson junction in a perpendicular magnetic field is represented by the modulus of the Fourier transform of the current density distribution in real space $J_s(x)$[9]. This means that one can extract the current density distribution of a JJ by calculating the inverse Fourier transform of the measured Fraunhofer pattern. For example, a homogenous current distribution will give the regularly studied Fraunhofer pattern, while more complex current distributions lead to more complex diagrams. Here we focus on the case of having edge states or just bulk contribution. The current distribution and the corresponding Fraunhofer pattern to different cases combining bulk and edge conduction are shown in Supplementary Fig. 13.

The process to calculate the Fraunhofer pattern from the real space current density distribution is done following the procedure developed by Dynes and Fulton[10]. Given a certain $J_s(x)$, its complex Fourier transform will yield the complex critical current function: $\mathfrak{I}_c(\beta) = \int_{-\infty}^{\infty} dx\, J_s(x) e^{i\beta x}$, where $\beta = (L+2\lambda)B/\Phi_0$ is a normalized field in units of µm, L is the length of the JJ and λ is the penetration depth into the superconducting electrodes. The experimentally measured I_c is then given by the modulus of this complex current density distribution $I_c(\beta) = |\mathfrak{I}_c(\beta)|$. In our case, we use the formalism given in section G about 2D JJs to calculate the

pattern. This means that instead of using $L+2\lambda$, we use the aproximation $w/1.8$ to account for the fact that the material will not screen the magnetic field.

In order to simulate the asymmetries observed in the data of device A we introduce some phase shifts into the patterns to simulate the magnetic origin of the signals. The phase shifts are introduced as an extra field in the β parameter by substituting $B = B_{ext} + \varphi$, where B_{ext} is the external field and φ will be the extra phase. This phase could have different origins, but in this model we just study it as a component which changes the effective field experienced by the sample. The next step is to separate these phases for the different components of the pattern by introducing φ_{edge1}, φ_{edge2} *and* φ_{bulk}, corresponding to the different edges and the bulk of the JJ. When $\varphi_{edge1} = \varphi_{edge2}$ we refer to it simply as φ_{edges}. Supplementary Fig. 14 shows Fraunhofer patterns calculated giving different phases acquired by the edges. In general we keep $\varphi_{bulk} = 0$, because we are interested in the asymmetries of the data. The bulk phase would contribute to a total shift of the pattern, but would not produce asymmetries. We find that asymmetries in $I_c(B)$ arise when $\varphi_{bulk} \neq \varphi_{edges}$, yielding a signal in which $I_c^{\pm}(B^+) \neq I_c^{\pm}(B^-)$, as we observe in the experiment (see Supplementary Fig. 14d). In order to obtain the current-field coupling leading to the tilt of the signal, the phase of the edges needs to change sign with the current direction: $\mathrm{sgn}(\varphi_{edges}(I^+)) = -\mathrm{sgn}(\varphi_{edges}(I^-))$. This implies that the current carries an effective field which is opposite for opposing current directions. By introducing this current induced phase, we obtain all the broken symmetries observed in the pattern of device A, this is: $I_c^{\pm}(B^+) \neq I_c^{\pm}(B^-)$ and $I_c^{\pm}(B^+) \neq I_c^{\mp}(B^+)$, while keeping the symmetry along the I - B coupled direction: $I_c^{\pm}(B^+) \neq I_c^{\mp}(B^-)$. In the experiment this symmetry is further broken due to the hysteresis. This could be broken in our model just by adding an extra phase to the whole device, which would shift the pattern to the positive or negative field direction.

From this model we can conclude that the experimental Fraunhofer of Fig. 2a requires the presence of edge states which carry an additional phase with respect to the bulk, this phase changes sign for a given current direction and, in addition, there is a general magnetism in the junciton which is responsible for the global phase shift and the hysteresis at low T. As was discussed in the main text, all these ingredients can be explained in the framework of a valley polarized state, in which the current can switch the orbital magnetic state. Finally, the fact that $\varphi_{edge1} \neq \varphi_{edge2}$ as in Supplementary Fig. 14f, can be understood in terms of having at least two different valley polarized domains which would effectively give a different phase for the two edges. Comparing Supplementary Fig. 14f to the data of Supplementary Fig. 14a, this model resembles the most the measured data and therefore is chosen to be plotted in Fig. 2b of the main text.

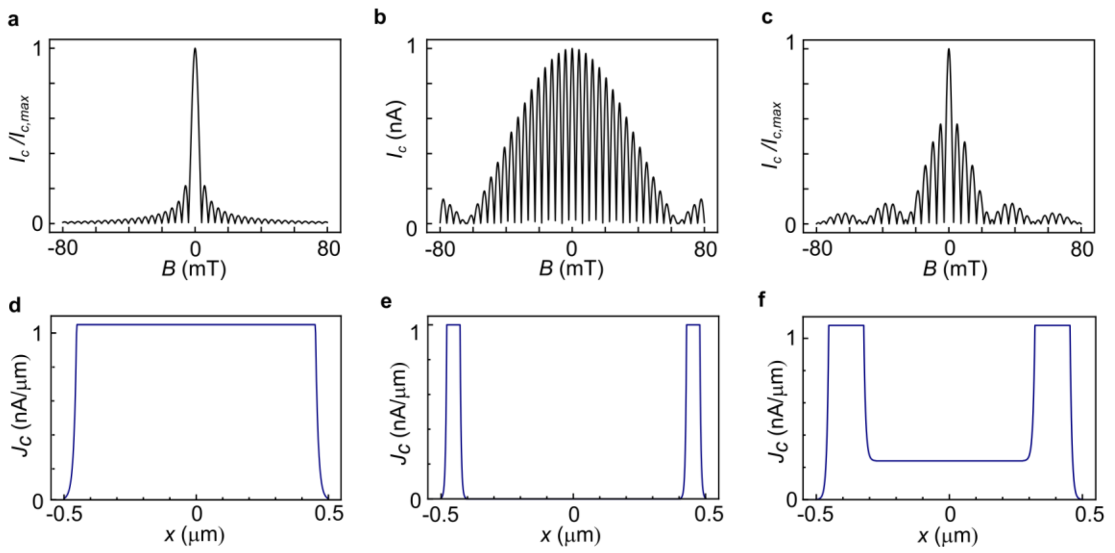

Supplementary Fig. 13. Fraunhofer patterns corresponding to different current density distributions. a-c, Normalized critical current $I_c/I_{c,max}$ vs. perpendicular magnetic field B corresponding to the current density J_c distributions shown in **d-f**, where x is the width of the JJ.

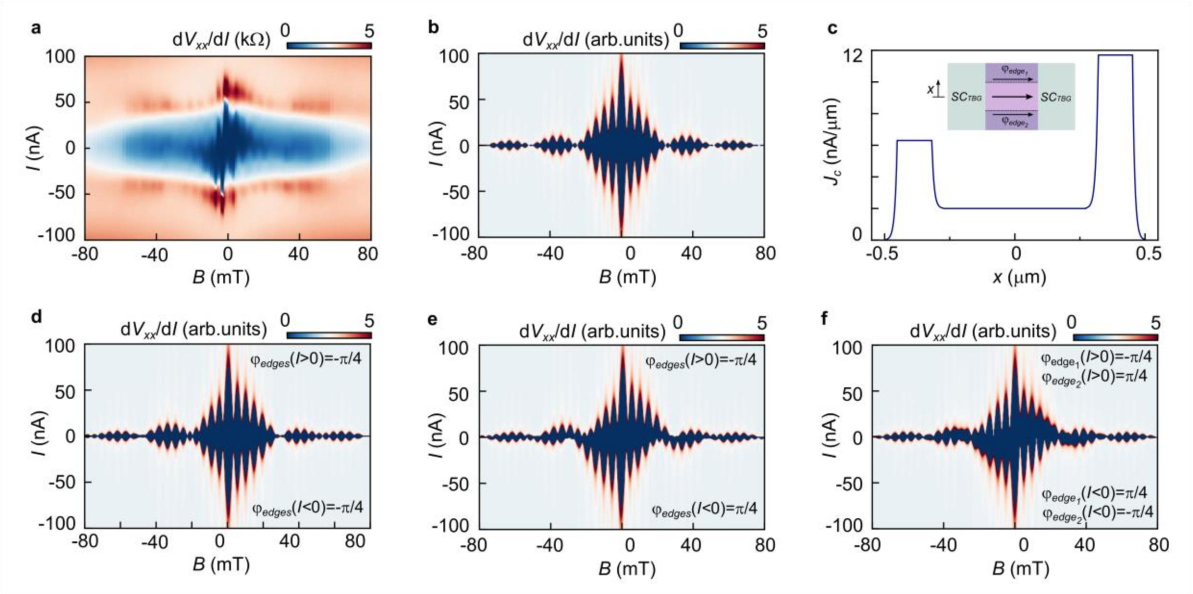

Supplementary Fig. 14. Asymmetric Fraunhofer patterns corresponding to different symmetry broken current densities. a, Fraunhofer pattern to higher fields at the SC/CI'/SC position. **b,** Modelled Fraunhofer pattern based on the current density profile of **c,** Current density profile to calculate the Fraunhofer patterns including both bulk and edge contribution. The inset shows a cartoon displaying the superconducting TBG forming the JJ (SC_{TBG}) in light green and the weak link with bulk and edge contribution in purple and violet, as well as the different phases carried by the edges $\varphi_{edge,n}$, where n = 1, 2 for the top and bottom edge respectively. **d,** Calculated Fraunhofer pattern having a different phase between edge and bulk but with the same phase between edges. **e,** Same as **d**, but the phase of the edges changes when inverting the current direction. **f,** The phases of the two edges are opposite and they change sign upon changing the current direction.

I. Disregarding possible artifacts of the magnetic signals

A key question to answer when magnetic hysteresis and shifts from zero-field are observed in experiments is whether they can have a trivial origin, i.e. trapped flux in the magnet. It is known that superconducting magnets can trap flux in their coils, leaving fields of few mT when the magnets sit at the measured zero-field. This can also lead to trivial magnetic field hysteresis, as the trapped flux will depend on the magnet's history, leading to positive field shifts when previous field was negative and vice versa. For example, ramping from positive field will in general lead to the measured zero-field be present at a negative field position.

The first question is whether the shift of the Fraunhofer pattern measured at 800 mK in Fig. 3b could be just due to this trapped flux. In the following we argue why this cannot be the case. This Fraunhofer pattern was measured after having applied a B_M = +500 mT, followed by the sequence of (+15 mT→-15 mT) and then (-15 mT→+15 mT), in order to extract the hysteresis at that field. In this case, the maximum critical current values in both the B_{up} and B_{dn} patterns are shifted towards positive fields, but no hysteresis is observed, as is seen in Fig. 4d. This behavior is not in line with the trapped flux scenario, since in that case we should either have two different shifts (from the up and down sequence). However, we observe a shift to positive fields, which we attribute to the polarized orbital magnet after having applied +500 mT. The shift of +2.5 mT is in line with the expected shift from the orbital magnetization as is explained in the main text.

The second question is whether the measured hysteresis at low *T* could be due to the magnet. In this case the *T* dependence of the hysteresis should be enough to disregard an origin due to trapped flux in the magnet. While the sample *T* is increased from 35 mK to 800 mK, the magnet's temperature is kept constant at ∼3.6 K. Therefore, all the features seen in Fig. 4 and Supplementary Fig. 9 are only due to the sample *T* increase and cannot be related to the magnet's trapped flux.

We would like to note that we do see some shifts in patterns which could be attributed to the magnet. For example, Fig. 1e (left) has a shift of -2 mT. Although we cannot conclude the origin of this shift, in this case it could be due to the trapped flux since the pattern is shifted to negative fields and was measured coming from a large positive field of +150 mT. However, it is important to note that such shifts are not always observed. For example, Fig. 1e (center) was measured from -200 mT and shows a shift ∼+0.2 mT. Therefore, we want to emphasize that the hysteresis of Fig. 4 is not related to this and has a clear distinct origin. Although we could expect some small shifts from 0 field due to some trapped flux, the *T* dependence evolution, the disappearance of phase jumps and the asymmetry after overcoming a coercive field as shown in Supplementary Fig. 10 cannot be explained by this.

J. The model Hamiltonian for the TBG based-Josephson junction

In this section, we present the model Hamiltonians for this Josephson junction, which will be used to calculate the Fraunhofer patterns. The Josephson junction studied in this experiment is rather special in the sense that all parts are formed by the MATBG only. As schematically plot in Supplementary Fig. 15a, this Josephson junction consists of three parts: the left and right parts are the superconducting MATBG which conducts supercurrents, and the middle part that links the two superconducting parts is also formed by a MATBG but being closed to half-filling, where correlated insulating phases are known to be established.

To capture the bands of MATBG, we used a hexagonal lattice model[11,12], which is written as:

$$H_0 = \sum_{j,\boldsymbol{\delta}^{(1)}} (\mathrm{c}_j^\dagger \cdot \hat{T}_1 \cdot \mathrm{c}_{j+\boldsymbol{\delta}^{(1)}} + \mathrm{H.c.}) + \sum_{j,\boldsymbol{\delta}^{(3)}} (\mathrm{c}_j^\dagger \cdot \hat{T}_3 \cdot \mathrm{c}_{j+\boldsymbol{\delta}^{(3)}} + \mathrm{H.c.}). \quad (1)$$

where the $c_j = (c_{j+,\uparrow}, c_{j+,\downarrow}, c_{j-,\uparrow}, c_{j-,\downarrow})$ denotes a four-component electron annihilation operator with $c_{j\pm}$ being the annihilation operator for $p_x \pm ip_y$ orbitals, ↑/↓ representing spin up/down at a site $\boldsymbol{j}$, and the first-nearest neighbor and the fifth-nearest neighbor (to break the emergent SU(4) symmetry) hopping terms are considered with hopping matrix $\hat{T}_1 = [t_1, 0; 0, t_1] \otimes \sigma_0$, $\hat{T}_3 = [t_2, 0; 0, t_2^*] \otimes \sigma_0$, the connecting vectors $\boldsymbol{\delta}^{(1)} = \{\boldsymbol{\delta}_1, C_3\boldsymbol{\delta}_1, C_3^2\boldsymbol{\delta}_1\}$, $\boldsymbol{\delta}^{(3)} = \{\boldsymbol{\delta}_3, C_3\boldsymbol{\delta}_3, C_3^2\boldsymbol{\delta}_3\}$ (see Supplementary Fig. 15b), σ_0 is an identity matrix defined in spin-space. This model respects D_3 point group symmetry, spin $SU(2)$ symmetry, and the orbital $U(1)$ symmetry. It is also worth noting that we can regard the two orbitals $p_x + i\xi p_y$ as a representation of two valleys[11] $\xi = \pm$. In the calculations, we adopted the model parameters from ref. [11], where $t_1 = 0.331$ meV, $t_2 = -0.01 + 0.097i$ meV.

Based on the insight about the presence of the spin and orbital magnetism, we write the model Hamiltonian of this Josephson junction formed by the MATBG as:

$$H = H_0 + H_h + H_{mu} + H_{order}. \quad (2)$$

where H_0 captures the bands of MATBG as written in Eq. (1). Let us introduce the details for other terms as follows.

To enable the bands to form orbital Chern bands, we added the Haldane Hamiltonian[13]:

$$H_h = \sum_{j\in A,\boldsymbol{\delta}^{(2)}} [(\mathrm{c}_j^\dagger \cdot \hat{T}_2 \cdot \mathrm{c}_{j+\boldsymbol{\delta}^{(2)}} + \mathrm{H.c.}) + m_{stag}\mathrm{c}_j^\dagger \cdot \mathrm{c}_j] + \sum_{j\in B,\boldsymbol{\delta}^{(2)}} [\mathrm{c}_j^\dagger \cdot \hat{T}_2^\dagger \cdot \mathbf{c}_{j+\boldsymbol{\delta}^{(2)}} + \mathrm{H.c.} - m_{stag}\mathrm{c}_j^\dagger \cdot \mathrm{c}_j], \quad (3)$$

where the next-nearest connecting vectors $\delta^{(2)} = (\boldsymbol{\delta}_2, C_3\boldsymbol{\delta}_2, C_3^2\boldsymbol{\delta}_2)$, A and B are the sublattice indices, the complex hopping matrix $\hat{T}_2 = [t_f e^{i\varphi}, 0; 0, t_f e^{-i\varphi}] \otimes \sigma_0$ and m_{stag} is a staggered potential. Without loss of generality, we set $t_f = 0.2t_1$, $m_{stag} = 0.1t_1$, $\varphi = \pi/2$, where the bands are topological [3]. This consideration is motived by another experiment with the same experimental setup, where orbital moiré Chern bands were found[14]. Noted what is esstential in our discussions is that the bands are orbital Chern bands carrying net orbital magnetism. In this sense, a phenomenological term as Eq. (3) is sufficient for our purpose. Physically, these terms can be induced by the combination of the moiré potential and some C_2T breaking terms. Note although we added the Haldane Hamiltonian for the whole MATBG, it is only crucial for the middle part of the junction to generate the orbital magnetism upon valley polarization. We checked whether the Haldane Hamiltonian is added or not in the left and right superconducting

part will not affect the features of the Fraunhofer pattern as long as the chemical potential is far from the charge neutrality point.

The filling difference between different regions is captured by H_μ:

$$H_\mu = -\sum_{j\in\mathbf{L},\mathbf{R}} \mu_{sc} c_j^\dagger \cdot c_j - \sum_{j\in\mathbf{M}} \mu c_j^\dagger \cdot c_j \quad (4)$$

with μ_s, μ respectively, denotes the chemical potential of the superconducting MATBG parts in the left, right part (label as **L**, **R**) of the junction, and the MATBG near half-filling in the middle part (label as **M**) of the junction (Supplementary Fig. 15a). In the calculation, we fix $\mu_s = -0.5$ meV in the superconducting part which is around the middle of moiré bands.

Finally, to capture the phase orders in different parts, we introduced a mean-field Hamiltonian H_{order}, which is written as:

$$H_{order} = \sum_{j\in\mathbf{L},\mathbf{R}} \left(|\Delta_{sc}| e^{i\phi_{L(R)}} c_j^\dagger (\tau_x \otimes i\sigma_y) c_j^\dagger + \mathrm{H.c.}\right) + \sum_{j\in\mathbf{M}} c_j^\dagger (\Delta_{sp}\tau_0 \otimes \sigma_z + \Delta_{vp}\tau_z \otimes \sigma_0) c_j. \quad (5)$$

where Pauli matrices τ, σ operates on orbital-, spin-space, respectively, Δ_{sc}, Δ_{sp} and Δ_{vp} denotes the order parameters of superconducting states, spin-polarized states, valley-polarized states, respectively. The first term captures the superconducting phase $\phi_{L(R)}$ of the left (right) part of the junction. Here, we assumed the superconducting order parameter takes the conventional BCS form, which is most likely to be stabilized by the electron-phonon interaction[15,16]. Considering that the key features of the Fraunhofer patterns arise from the spin and orbital magnetism in the middle part, similar Fraunhofer patterns may be found even when the paring is unconventional, such as p-wave or d-wave paring, although some details such as the amplitude of supercurrent should be different. A detailed study of the junction in the presence of both unconventional pairings and magnetism is beyond the scope of this work, but in general this is interesting and we leave it as future work. The second term captures the spin- and valley-polarized states that generate the spin and orbital magnetism in the middle part of the junction.

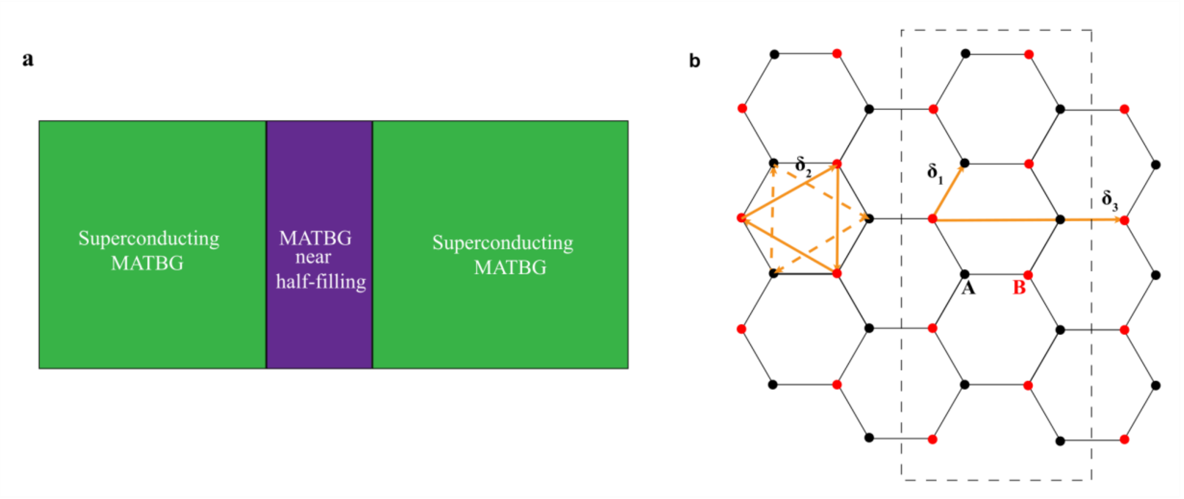

Supplementary Fig. 15. Sample and lattice geometry considered in the theoretical calculation for this MATBG based-Josephson junction. **a,** A schematic plot of the studied twisted bilayer graphene based Josephson junction. **b,** A figure to illustrate the effective hexagonal

lattice for the Fraunhofer pattern calculations, where δ_j are bond vectors, A and B label two different sub-lattices, the yellow arrow indicates there is a hopping between two sites. The dash lines highlight the shape of the recursive unit in our Fraunhofer pattern calculations.

K. The method for the calculation of the Fraunhofer patterns

After introducing the model Hamiltonian for the Josephson junction formed by the MATBG, we now present the detailed process for the calculation of Fraunhofer patterns. To evaluate the Fraunhofer patterns, we need to take account into the effects of external magnetic fields $\mathbf{B}$ on the junction, where we consider $\mathbf{B}$ is finite only in the middle part. This is done by replacing the hopping terms in the middle part with the Peierls substitution as:

$$t_{jj'} \to t_{jj'} e^{i\frac{e}{\hbar}\int \mathbf{A}(\mathbf{r})\cdot d\mathbf{r}}. \tag{6}$$

The Landau gauge fields are taken as $\mathbf{A}(\mathbf{r}) = (0, Bx, 0)$, where we set x-direction to be the transverse direction of the junction. Let us insert $\mathbf{A}(\mathbf{r})$ back to Eq. (6) and we can obtain

$$t_{jj'} e^{i\frac{e}{\hbar}\int \mathbf{A}(\mathbf{r})\cdot d\mathbf{r}} = t_{jj'} e^{i\frac{2\pi\bar{x}\Delta y}{\sqrt{3}a^2 N_t}\frac{\Phi}{\Phi_0}}. \tag{7}$$

Here, a is the lattice constant, N_t is the total number of hexagonal plaquettes, the flux quantum $\Phi_0 = h/2e$, the total flux $\Phi = \frac{3}{2}N_t B a^2$, $\bar{x} = (x_1 + x_2)/2, \Delta y = y_2 - y_1$ with $\boldsymbol{j} = (x_1, y_1), \boldsymbol{j}' = (x_2, y_2)$.

After this Peierls substitution, we can evaluate the Josephson current using the lattice recursive Green's function method[17,18]. Here, the recursive unit is shown in Supplementary Fig. 15b (within dashed lines), which is formed by two zigzag chains. The specific recursive processes are:

(i) obtain the edge Green's function of the superconducting MATBG in the right and left part of Josephson junction by recursively calculating:

$$G_{ll}^{L(R)}(\omega_n) = (i\omega_n - H_{ll}^{L(R)} - \Sigma_{l,l}^{L(R)}(\omega_n))^{-1}, \tag{8}$$

$$\Sigma_{l,l}^{L} = V_{l,l-1} G_{l-1,l-1}(\omega_n) V_{l,l-1}^{\dagger},\ \Sigma_{l,l}^{R} = V_{l,l+1} G_{l+1,l+1}(\omega_n) V_{l,l+1}^{\dagger}. \tag{9}$$

where l is the index for the recursive unit, $\omega_n = (2n+1)\pi T$ is the Matsubara frequency with T as temperature. Here the intra-unit Green's function $G_{ll}(\omega_n)$ and self-energy $\Sigma_{l,l}$ is evaluated iteratively until $G_{ll}(\omega_n)$ is saturated using the intra-unit Hamiltonian $H_{ll}^{L(R)}$ of the left and right part, the hopping Hamiltonian $V_{l+1,l}$ between the unit l and $l+1$. Note that $H_{ll}^{L(R)}$ and $V_{l+1,l}$ are Bogoliubov de Gennes (BdG) Hamiltonians defined in Nambu basis $(c_j, c_j^{\dagger})$. For example, $V_{l+1,l} = \mathrm{diag}[V_{l+1,l}^{ee}; V_{l+1,l}^{hh}]$ with $V_{l+1,l}^{hh} = -V_{l+1,l}^{ee*}$. Also note the initial Green's function $G_{00}(\omega_n) = (i\omega_n - H_{ll}^{L(R)})^{-1}$, and the superconducting phase in H_{ll}^{L} and H_{ll}^{R} are ϕ_L and ϕ_R, respectively. After these iterations, we can obtain the intra-unit Green's function $G_{l_1,l_1}(\omega_n)$ and $G_{l_3,l_3}(\omega_n)$, where l_1 (l_3) is the index of the first (last) unit of the middle part.

(ii) obtain the intra-unit Green's function $G_{l_2 l_2}(\omega_n)$, nearest-unit Green's function $G_{l_2,l_2+1}(\omega_n)$, where l_2 labels a unit cell in the middle part. To obtain this, we can first perform a similar recursive calculation as step (i) for the l_1 to $l_2 - 1$ and for l_3 to $l_2 + 1$ part, i.e.,

$$G_{ll}(\omega_n) = (i\omega_n - H_{ll}^M - \Sigma_{ll}(\omega_n))^{-1}, \tag{10}$$

$$\Sigma_{l+1,l+1} = V_{l+1,l} G_{ll}(\omega_n) V_{l+1,l}^{\dagger}. \tag{11}$$

Here initial Green's function for l_1 to $l_2 - 1$ and l_3 to $l_2 + 1$ part are G_{l_1,l_1}^L and G_{l_3,l_3}^R . In this way, we can obtain $\Sigma_{l_2-1,l_2-1}(\omega_n)$ and $\Sigma_{l_2+1,l_2+1}(\omega_n)$. Then we can obtain

$$G_{l_2,l_2}(\omega_n) = (i\omega_n - H_{ll}^M - \Sigma_{l_2-1,l_2-1}(\omega_n) - \Sigma_{l_2+1,l_2+1}(\omega_n))^{-1}, \tag{12}$$

$$G_{l_2,l_2+1}(\omega_n) = G_{l_2+1,l_2+1}(\omega_n) V_{l_2,l_2+1}^{\dagger} G_{l_2,l_2}(\omega_n), \tag{13}$$

$$G_{l_2+1,l_2}(\omega_n) = G_{l_2,l_2}(\omega_n) V_{l_2,l_2+1} G_{l_2+1,l_2+1}(\omega_n). \tag{14}$$

(iii) evaluate the Josephson current:

$$I(\Delta\phi) = -iT \sum_n \mathrm{Tr}[\tilde{V}_{l_2,l_2+1} G_{l_2+1,l_2}(\omega_n) - \tilde{V}_{l_2,l_2+1}^{\dagger} G_{l_2,l_2+1}(\omega_n)], \tag{15}$$

where the current depends on the phase difference $\Delta\phi = \phi_L - \phi_R$ and in particular, being distinct from $V_{l+1,l}$, the $\tilde{V}_{l+1,l} = \mathrm{diag}[V_{l+1,l}^{ee}; -V_{l+1,l}^{hh}]$, i.e., there is an additional sign in the hole part, as the electron and hole carry opposite currents. More details of this method can be referred to ref. [17,18].

L. Orbital Chern bands and the orbital magnetic moment.

In this section, we illustrate the features of energy bands of normal states in our model Hamiltonian. The energy bands along high symmetry lines with and without valley polarization are shown in Supplementary Fig. 16a and Supplementary Fig. 16b. Supplementary Fig. 16a reproduces the bands of MATBG given in ref. [11,12], where the red lines and green lines label the bands from two valleys, respectively. In Supplementary Fig. 16b, we added a valley polarization $\Delta_{vp} = 1.5$ meV and the Haldane terms, but leaves the spin polarization to be zero so that each band is doubly degenerate. Here the degeneracy at $\overline{K}$ is lifted by the Haldane terms, which break C_2T symmetry, and near half-filling υ = -2, an isolated band from one valley would be occupied as a result of the valley polarization. Since the cooper pairs are pairing states from intervalley, the supercurrent will be weakened stronger with the increase of valley-polarization strength Δ_{vp}.

Another crucial feature is that the bands are topological nontrivial now, i.e. they are orbital Chern bands, due to the Haldane terms (see Eq. 3). To illustrate this, the distribution of Berry curvature in the Brillouin zone is depicted in Supplementary Fig. 16c. It clearly shows the band carries a finite Chern number $C = 1$. Note the Chern numbers C of Chern bands in each valley carry the same sign but are opposite for opposite valleys (Suppleementary Fig. 16b), as the two valleys are related by the time-reversal operation. As we mentioned, the appearance of orbital Chern bands in these MATBG samples near half-filling have been found in ref. [[14]] already.

An important consequence of the orbital Chern bands is to give rise to orbital magnetism[19,20]. To show this explicitly here, we calculated the orbital magnetic moment $m_{n\mathbf{k}}$ carried by these orbital Chern bands with[19–22]:

$$m_{n\mathbf{k}} = \frac{e}{2\hbar}\sum_{l\neq n} \mathrm{Im}\frac{<u_{n\mathbf{k}}|\nabla_{\mathbf{k}}H(\mathbf{k})|u_l(\mathbf{k})>\times<u_{l\mathbf{k}}|\nabla_{\mathbf{k}}H(\mathbf{k})|u_{n\mathbf{k}}>}{E_{l\mathbf{k}}-E_{n\mathbf{k}}}, \quad (16)$$

where $H(\mathbf{k})|u_{n\mathbf{k}} >= E_{n\mathbf{k}}|u_{n\mathbf{k}} >$, l, n are band indices. To see the magntude of the orbital magnetic moment of per electron for n-th band $M_{n,orbit}$, we can calculate:

$$M_{n,orbit} = \frac{1}{N}\sum_{\mathbf{k}} m_{n\mathbf{k}}, \quad (17)$$

where N is the number of k points. We found the calculated $M_{n,orbit}$ is about 3 μ_B for each moire band according to the orbital magnetic moment distribution shown in Supplementary Fig. 16d. In the next part, we will show such a large orbital magnetic moment will give rise to a shift in the Fraunhofer pattern being compable to the experiment.

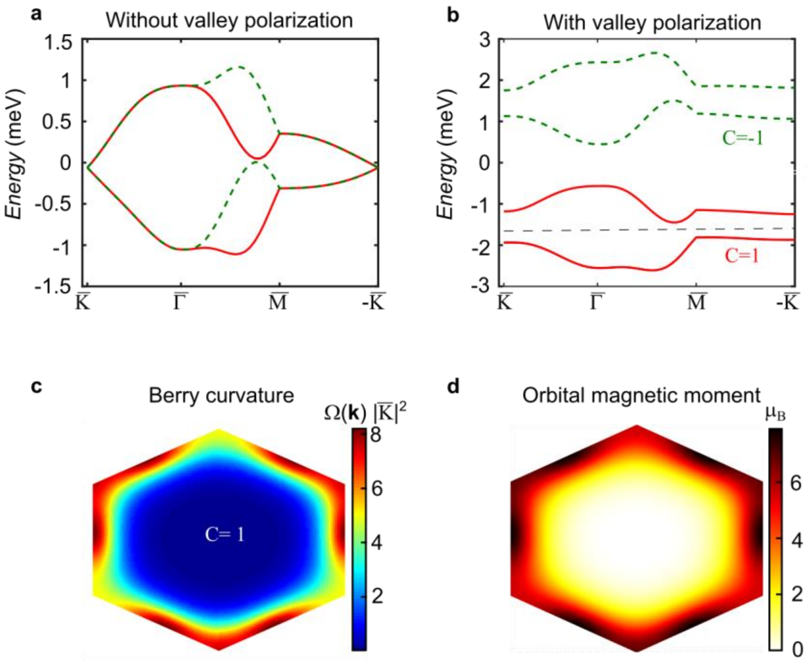

Supplementary Fig. 16: a-b, Band structures of MATBG with and without valley polarization. The solid red bands are from one valley, while the dashed green bands are from the opposite valley. In **b** the Haldane term is added to lift the degeracies at $\pm\overline{K}$ and a dashed black line is used to indicate the half-filling position, where there is an insulating gap. **c,** Distribution of Berry curvature of the lowest bands in **b** in the Brillouin zone, which clearly shows a Chern number $C = 1$. **d,** Orbital magnetic moment in k-space in units of Bohr magneton μ_B of the lowest band in **b**.

M. Estimate the phase shift in Fraunhofer patterns arising from the presence of net magnetization

The magnetic field in a sample is:

$$B = \mu_0(H + M), \quad (18)$$

where H can be regarded as the applied magnetic fields, $\mu_0 = 4\pi \times 10^{-7}$ T$\cdot$ m/A is the vacuum magnetic permeability, and the magnetization, including the orbital and spin part, is given by:

$$M = M_{orbit} + M_{spin}. \tag{19}$$

Let us assume each electron carries γu_B magnetic moment, where $u_B = e\hbar/2m_e = 9.27 \times 10^{-24}$J/T, and denote the electron density is ρ m $^{-3}$. The magnetization thus is written as:

$$M = \gamma\rho u_B. \tag{20}$$

Therefore, the effective B field from the magnetization is:

$$B_{eff} = \mu_0 M = \gamma\rho \times 1.13 \times 10^{-29}\text{T}. \tag{21}$$

For the twisted bilayer graphene near the magic angle, the moire pattern lattice constant is $L_M = 14$nm and the thickness is about 0.34 nm. Near the half-filling $\nu = -2$, the electron density (in unit of m^{-3}) is:

$$\rho = 2/(14^2 \times \sqrt{3}/2 \times 0.34) \times 10^{27} \approx 3.47 \times 10^{25} \tag{22}$$

We can also use the experimental carrier density $\sim 1.72 \times 10^{12}$ cm $^{-2}$, and the electron density becomes:

$$\rho \approx 1.72/0.34 \times 10^{25} = 5.07 \times 10^{25}. \tag{23}$$

Using the estimated electron density, we obtain the shift fields due to the presence of magnetism as:

$$B_{eff} \approx 0.4 \sim 0.6\, \gamma \text{ mT} \tag{24}$$

There are two moire bands and each moire band carries a orbital magnetic moment of $3\mu_B$ according to the previous section so that $\gamma \sim 6u_B$ for the valley polarized state at $\nu = -2$. As a result, the shifting field B_M is eastimated to be 2.4~3.6 mT, being consistent with the observed 2.5 mT phase shift in the Fraunhofer pattern. After adding this shifting field, the peak of Fraunhofer pattern will be moved to a finite field, as illustrated in Supplementary Fig. 17b.

N. Details for the theoretically calculated Fraunhofer patterns

In this section, we summarize some details for the theorectically calculated Fraunhofer patterns. To obtain the critical current as a function of Φ, i.e., Fraunhofer pattern, at each external field, we vary the phase different over 0 to 2π and obtain the largest positive and smallest negative current as $I_c^+(\Phi)$ and $I_c^-(\Phi)$. In our calculations, we set pairing gap $\Delta =$ 0.1 meV, and we tuned μ, which effectively changes the filling, and the order parameter in the middle region: Δ_s and Δ_{vp}, which give rise to the spin and orbital magnetism.

First, we set μ in the middle of the bands and did not add the valley polarization. In this case, the middle region behaves as a normal metal and the whole junction is a SC-N-SC

junction. As a result, we obtained the standard Fraunhofer pattern, as shown in Supplementary Fig. 17a, where $I_c \sim |\frac{\sin(\pi\Phi/\Phi_0)}{\pi\Phi/\Phi_0}|$.

Next, we turn on the valley polarization Δ_{vp}. As we discussed, the valley polarization has several effects: (i) As we consider the inter-valley pairing, the valley polarization would suppress the formation of the cooper pairing and reduce the supercurrent through this junction; (ii) The valley polarization will break the time-reversal symmetry and lift the valley degeneracy. Consequently, the combination of orbital Chern bands and valley polarization results in net orbital magnetism. Especially, as we showed, the orbital magnetic moment can be tens of Bohr magneton and leads a shift in Fraunhofer pattern with a shift flux $\Phi_M = B_{eff}S$ (as illustrated in Supplementary Fig. 17b), S is the junction area. On the other hand, we found the Fraunhofer pattern with the valley polarization Δ_{vp} typically follows a standard Fraunhofer pattern when μ is away from half-filling, being expected for an SC-I-SC junction or an SC-N-SC junction (I denotes insulator).

However, we found the Fraunhofer pattern deviates from the standard Fraunhofer pattern when the μ is closed to the half-filling, and especially, some asymmetric behaviors are established. The calculated Fraunhofer pattern near half-filling with $\Delta_{vp} = 0.8$ meV, $\mu = -0.9$ meV is plotted in Supplementary Fig. 17c. Here we chose a sizable valley polarization so that the two valleys are fully separated. It can be seen that the Fraunhofer pattern exhibits a similar asymmetric behavior as observed in the experiment. We also checked the asymmetric behaviour is quite robust as long as the Δ_{vp} can polarize two valleys considerably, as shown in Supplementary Fig. 17d-i, where we display the Fraunhofer patterns at half-filling for various Δ_{vp}.

However, we found the asymmetry with respect to B fields is lost when the Haldane terms are removed, regardless of the strength of valley polarization (Supplementary Fig. 17d). This suggested the orbital magnetic moment plays a crucial role in giving rise to the asymmetric behaviour. This understanding is also reasonable as the magnetic fields couple with the orbital moment and can shift the Chern bands. Because this shifting is opposite for opposite fields (the applied fields are small~several mT would not reverse the sign of orbital moment), it thus could result in different critical supercurrent through this junction for opposite fields. On the other hand, the orbital magnetic moment gradually concentrates near half-filling, and thus we obtained a relatively symmetric Fraunhofer pattern in calculation when μ is artifically tuned away from half-fillings. Therefore, our interpretation that this asymmetry with respect to B in Fraunhofer patterns arises from the coupling between external B fields and orbital magnetic moment of bands is qualitatively consistent with our calculations. This interpretation would also directly imply there is a current-induced magnetization switching in the experiment as discussed in the main text, since the asymmetric behaviour is reversed for opposite current directions.

Beyond the bands level, here we also give a mean-field level discussion on how the coupling between B fields and orbital magnetization can introduce asymmetric Fraunhofer patterns in this junction. We assumed the sign of valley polarization Δ_{vp} with the switch of current direction and the B fields couples with Δ_{vp} as this valley polarized state carries orbital magnetic moment. We can describe this switching with a phenomenological free energy as

$F_{free} = a\Delta_{vp}^2 + b\Delta_{vp}^4 + \lambda_1 \text{sign}(I)\Delta_{vp} - \lambda_2 B\Delta_{vp}$. As shown in the main text Fig. 3e, the sign of valley polarization depends on the current direction in this case. Moreover, we would like to point out the coupling between magnetic fields and valley polarization order, i.e., $B\Delta_{vp}$ term, in general, gives a larger Δ_{vp} at the positive B field region, and a smaller Δ_{vp} at the negative B-field region. As a result, this can result in another mechanism for the possible asymmetry in Fraunhofer patterns at positive B field and negative B field range. Note the coercive field B_c ~300 mT is much larger than the applied fields, so the field itself would not switch the valley polarization, and the change of Δ_{vp} due to the change of B field thus should be quite small. It is worth mentioning that in our calculation, we found a 0.01 meV change in Δ_{vp} is enough to enable the asymmetry induced by this mechanism to be seen. However, whether several mT B fields used in the experiment can cause a meaningful change in Δ_{vp}, such as large as 0.01 meV, would depend on the specific details, such as domain walls and interactions.

But regardless of different levels of interpretations, our experimental data and our calculations strongly suggest asymmetric symmetry Fraunhofer patterns indicate a net orbital magentization near $\nu = -2$ and it can be switched by the supercurrent. To our knowledge, the orbital magnetic moment-induced asymmetric Fraunhofer pattern has not been realized so far. We thus think a more qualitative study is quite desirable and would leave this as another work.

In device B, the system is much more insulating. The Fraunhofer pattern is found to be almost purely SQUID-like, which implies the edge states are dominant near the Fermi energy. Here, we present a theoretical calculation to show the SQUID Fraunhofer pattern induced by the edge states at half-filling, in which the Chern number is equal to -2. We use the same model presented above, but the chemical potential is now set within the insulating gap at half-filling. Supplementary Fig. 18 displays the calculated Fraunhofer pattern with a valley polarization $\Delta_{vp} = -0.8$ meV, temperature $T = 0.02\Delta_s$. It is expected that the Fraunhofer pattern overall displays a SQUID feature in this case.

Finally, we discuss the feature of the Fraunhofer pattern in the presence of spin magnetism. As we discussed, the spin magnetism is one of the possibilities to give rise to the data in the low-temperature range ($T <$800 mK) according to the measured Fraunhofer patterns. To show explicitly, we calculated the Fraunhofer pattern by adding a spin polarization Δ_{sp}. Specifically, we inputted Δ_{sp} as a hysteresis loop of B field as shown in Supplementary Fig. 19a, where we define the saturated spin polarization Δ_{sp} as $\Delta_{saturate}$. In Supplementary Fig. 19c and Supplementary Fig. 19c, we show the Fraunhofer pattern with $\Delta_{saturate}/\Delta_{sc} = 0.5$ and $\Delta_{saturate}/\Delta_{sc} = 1$, Δ_{sc} is the superconducting gap. By sweeping the B-field from negative to positive and from positive to negative, we also found a hysteresis in the Fraunhofer pattern induced by the hysteresis of spin magnetism. This is due to the fact that the supercurrent carried by singlet Cooper pairings will be reduced by the increase of spin polarization. It thus would be also expected the hysteresis will be more prominent when the size of spin polarization characterized by $\Delta_{saturate}$ is comparable to the superconducting paring gap Δ_{sc}, as we showed in Supplementary Fig. 19c. This Fraunhofer pattern can reproduce all the main characteristics of the experimental measurements at 35 mK (Fig. 4c in the main text).

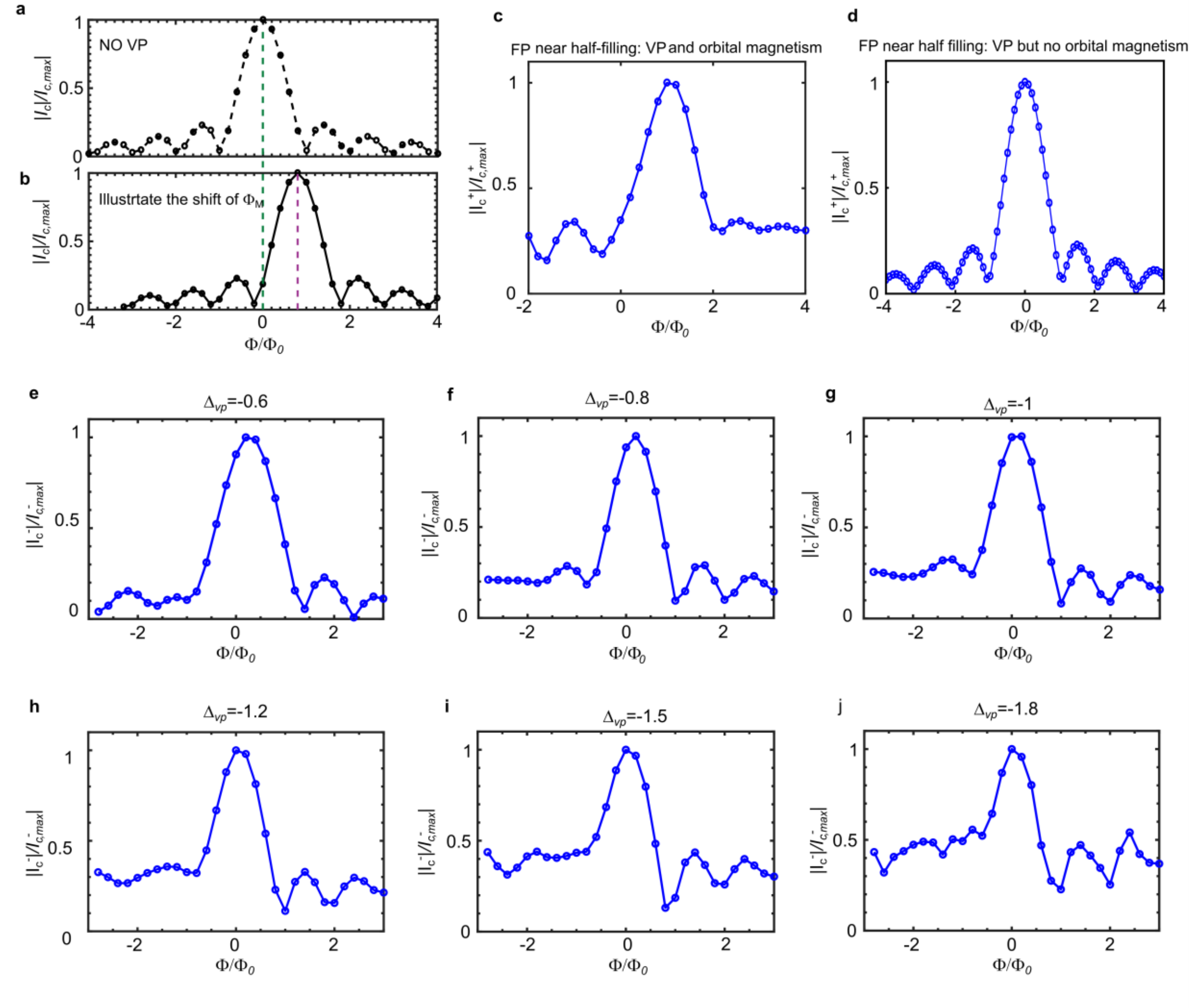

Supplementary Fig. 17: Fraunhofer pattern in the presence of valley polarization and orbital magnetism. **a,** Standard Fraunhofer pattern when the middle range is a metal, where I_c denotes the critical current. **b,** Shift of Φ_M in the Fraunhofer pattern due to the orbital magnetism. **c,** Typical Fraunhofer pattern near half-filling, which supports an insulating gap. The asymmetry induced by the valley polarization is quite clear. **d,** Fraunhofer pattern near half-filling “”with no *C2T* symmetry breaking terms. The asymmetry is not present in this case as there is no orbital magnetization. **e-j,** Calculated Fraunhofer pattern with various valley polarized strength Δ_{vp} (in units of meV) near half-filling. To compare with the experiment, we also add a shift of $+0.8\Phi_0$ for I_c^+ in the Fraunhofer pattern for **c**.

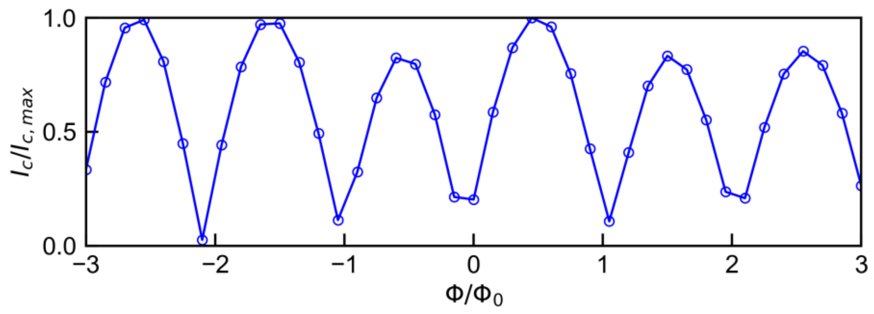

Supplementary Fig. 18. SQUID Fraunhofer pattern induced by edge states. The parameters are $\Delta_{vp}= -0.8$ meV, the temperature $T = 0.02\Delta_s$, and the chemical potential is set within the insulating gap at half-filling.

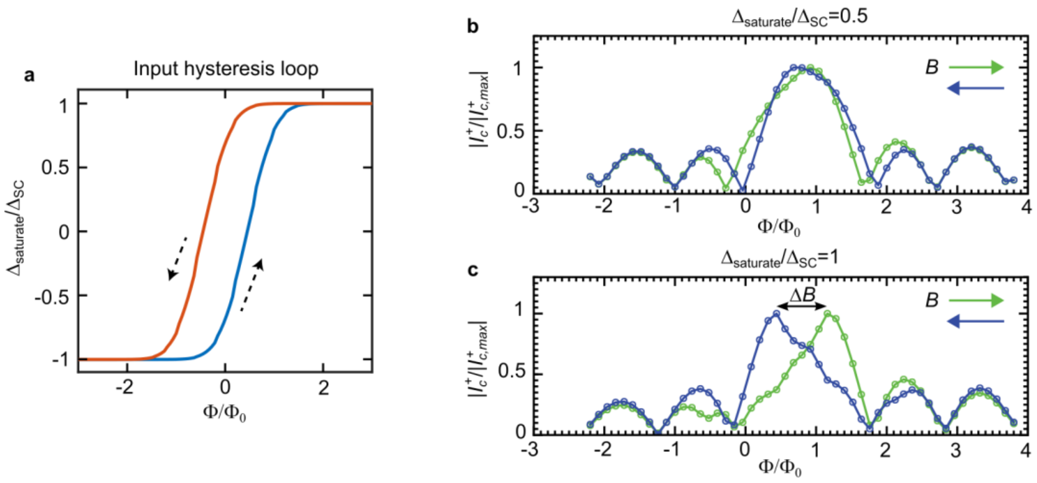

Supplementary Fig. 19. Hysteresis in Fraunhofer pattern from spin magnetism. **a,** Hysteresis loop inputted in the calculation, where $\Delta_{saturate}$ denotes the saturated spin polarization. **b,** Fraunhofer pattern with $\Delta_{saturate}/\Delta_{sc} = 0.5$. **c,** Fraunhofer pattern with $\Delta_{saturate}/\Delta_{sc} = 1$. ΔB signal the size of the hysteresis due to the spin polarization, as explained in the main text.

O. Fraunhofer pattern in the presence of unconventional pairings.

As we have pointed out in the main text, the unconventional Fraunhofer pattern, which only appears when the junction region is tuned near half-filling, should result from the correlated states near hallf-filling of the junction part instead of the superconducting part. Next, we demonstrate this point by showing the Fraunhofer patterns in the presence of various pairings. We first classify the possible pairings using irreducible representations of the D_3 crystal group of MATBG. For simplicity, we focus on all **k**-independent intervalley pairings. All the **k**-independent intervalley pairing forms in the spin and valley degree of freedom are summarized in Supplementary Table 1.

Next, we evaluate the Fraunhofer patterns by replacing the pairing matrix in Eq. (5) with these pairngs. The results are shown in Supplementary Fig. 20. Evidently, the resulting Fraunhofer patterns are not sensitive to the exact pairing forms, regardless of the spin-singlet pairing and spin-triplet pairing.

IRs	A_1	A_2	E
$C_{3z} = \tau_0 \otimes e^{-i\frac{\pi}{3}\sigma_z}$	$+1$	$+1$	$+2$
$C_{2y} = \tau_x \otimes i\sigma_y$	$+1$	$+1$	0
Spin-singlet	$\tau_x \otimes i\sigma_y$	—	—
Spin-triplet	$i\tau_y \otimes \sigma_x$	—	$(i\tau_y \otimes \sigma_z, i\tau_y \otimes \sigma_0)$

Supplementary Table 1: Classification of all possible momentum independent pairings of TBG according to the irreducible representations (IRs) of the D_3 symmetry group.

1) intervalley spin-singlet A_1 pairing : $\Delta_{A_1,s} = \tau_x \otimes i\sigma_y$,,

2) one one-dimensional intervalley spin-triplet A_1-pairing $\Delta_{A_1,t} = i\tau_y \otimes \sigma_x$,

3) one two-dimensional intervalley spin-triplet E-pairing with

E_1-pairing: $\Delta_{E,1} = i\tau_y \otimes \sigma_z$.

E_2-pairing: $\Delta_{E,2} = i\tau_y \otimes \sigma_0$.

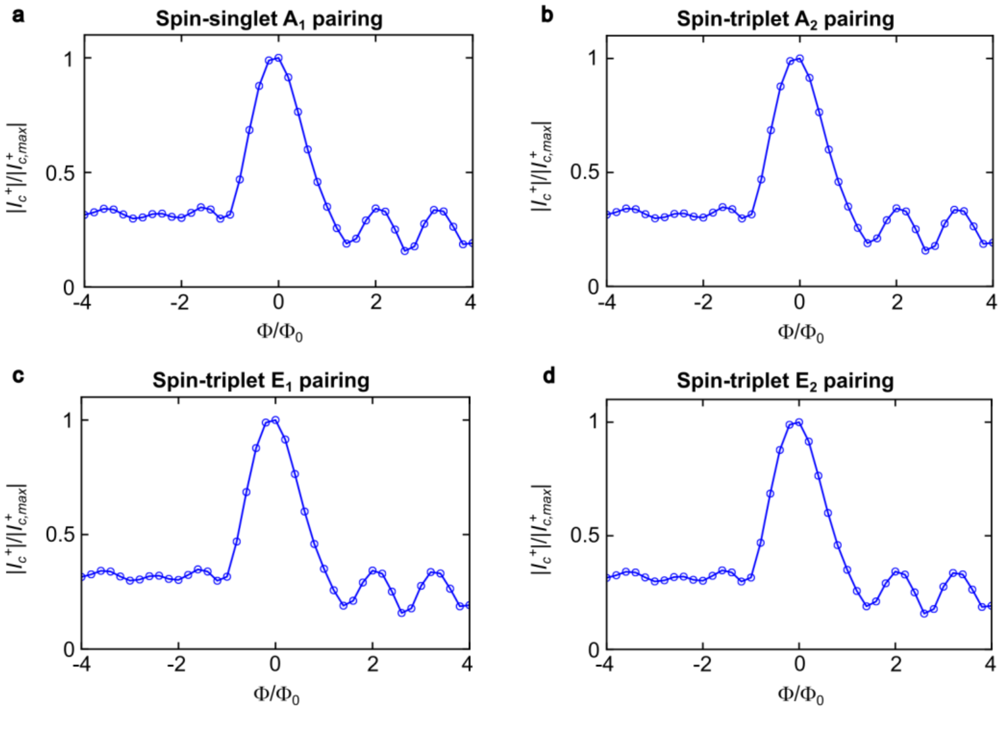

Supplementary Fig. 20. Fraunhofer pattern in the presence of various pairings. a-d, Display the Fraunhofer pattern for the spin-singlet A1 pairing, spin-triplet A1 pairing, spin-triplet E1 pairing and spin-triplet E2 pairing, respectively. Here we have set Δ_{vp} = -0.8 and other parameters to be the same as Supplementary Fig. 16c. This implies the form of order parameters is not a critical issue in studying the observed highly unconventional Fraunhofer patterns**.**

Supplementary References:

1. Wang, Y. T., Hu, Y. C., Chu, W. C. & Chang, P. Z. The fringe-capacitance of etching holes for CMOS-MEMS. *Micromachines (Basel)* **6**, 1617–1628 (2015).
2. Sadiku, M. N. O. *Numerical Techniques in Electromagnetics*. (CRC Press LLC, 2001).
3. Young, D. Iterative Methods for Solving Partial Difference Equations of Elliptic Type. *Trans Am Math Soc* **76**, 92 (1954).
4. Efetov, D. K. *et al.* Specular interband Andreev reflections at van der Waals interfaces between graphene and NbSe 2. *Nat. Phys.* **12**, 328–332 (2016).
5. Pearl, J. Current distribution in superconducting films carrying quantized fluxoids. *Appl. Phys. Lett.* **5**, 65–66 (1964).
6. Moshe, M., Kogan, V. G. & Mints, R. G. Edge-type Josephson junctions in narrow thin-film strips. *Phys. Rev. B - Condens. Matter Mater. Phys.* **78**, (2008).
7. Rodan-Legrain, D. *et al.* Highly tunable junctions and non-local Josephson effect in magic-angle graphene tunnelling devices. *Nat. Nanotechnol.* **16**, 769–775 (2021).
8. Thinkham, M. *Introduction to Superconductivity*. (McGraw-Hill, Inc., 1996).
9. Barone, P. & Paterno, G. *Physics And Applications Of The Josephson Effect*. (Wiley-Interscience, 1982).
10. Dynes, R. C. & Fulton, T. A. Supercurrent Density Distribution in Josephson Junctions. *Phys. Rev. B* **3**, 3015–3023 (1971).
11. Koshino, M. *et al.* Maximally Localized Wannier Orbitals and the Extended Hubbard Model for Twisted Bilayer Graphene. *Phys. Rev. X* **8**, 031087 (2018).
12. Yuan, N. F. Q. & Fu, L. Model for the metal-insulator transition in graphene superlattices and beyond. *Phys. Rev. B* **98**, 045103 (2018).
13. Haldane, F. D. M. Model for a quantum hall effect without landau levels: Condensed-matter realization of the 'parity anomaly'. *Phys. Rev. Lett.* **61**, 2015–2018 (1988).
14. Stepanov, P. *et al.* Untying the insulating and superconducting orders in magic-angle graphene. *Nature* **583**, 375–378 (2020).
15. Wu, F., Macdonald, A. H. & Martin, I. Theory of Phonon-Mediated Superconductivity in Twisted Bilayer Graphene. *Phys. Rev. Lett.* **121**, 257001 (2018).
16. Lian, B., Wang, Z. & Bernevig, B. A. Twisted Bilayer Graphene: A Phonon-Driven Superconductor. *Phys. Rev. Lett.* **122**, 257002 (2019).
17. Furusaki, A. DC Josephson effect in dirty SNS junctions: Numerical study. *Physica B: Physics of Condensed Matter* **203**, 214–218 (1994).
18. Asano, Y. Numerical method for dc Josephson current between d-wave superconductors. *Phys. Rev. B – Condens. Matter Mater. Phys.* **63**, 052512 (2001).

19. Liu, J., Ma, Z., Gao, J. & Dai, X. Quantum Valley Hall Effect, Orbital Magnetism, and Anomalous Hall Effect in Twisted Multilayer Graphene Systems. *Phys. Rev. X* **9**, (2019).

20. He, W. Y., Goldhaber-Gordon, D. & Law, K. T. Giant orbital magnetoelectric effect and current-induced magnetization switching in twisted bilayer graphene. *Nat. Commun.* **11**, 1–8 (2020).

21. Xiao, D., Chang, M.-C. & Niu, Q. Berry phase effects on electronic properties. *Rev. Mod. Phys.* **82**, 1959–2007 (2010).

22. Ceresoli, D., Thonhauser, T., Vanderbilt, D. & Resta, R. Orbital magnetization in crystalline solids: Multi-band insulators, Chern insulators, and metals. *Phys. Rev. B - Condens. Matter Mater. Phys.* **74**, (2006).
